# Removal of totally implanted venous access ports for suspected infection in the intensive care unit: a multicenter observational study

**DOI:** 10.1186/s13613-018-0383-9

**Published:** 2018-03-27

**Authors:** Marie Lecronier, Sandrine Valade, Naike Bigé, Nicolas de Prost, Damien Roux, David Lebeaux, Eric Maury, Elie Azoulay, Alexandre Demoule, Martin Dres, on behalf of the GrrrOH (Group for Research in Respiratory Intensive Care Onco-Hematology)

**Affiliations:** 1 Service de Pneumologie et Réanimation Médicale (Département “R3S”), Groupe Hospitalier Pitié-Salpêtrière Charles Foix, Assistance Publique-Hôpitaux de Paris, 75013 Paris, France; 2 Service de Réanimation médicale, Groupe Hospitalier Saint-Louis – Lariboisière – Fernand-Widal, Assistance Publique-Hôpitaux de Paris, Paris, France; 3 Service de Réanimation médicale, Groupe Hospitalier Est Parisien, Hôpital Saint-Antoine Paris, Assistance Publique-Hôpitaux de Paris, Paris, France; 4 Service de Réanimation médicale, Groupe Hospitalier Henri Mondor, Assistance Publique-Hôpitaux de Paris, Créteil, France; 5 Service de Réanimation médico-chirurgicale, Groupe Hospitalier Paris Nord, Hôpital Louis-Mourier, Assistance Publique-Hôpitaux de Paris, Colombes, France; 6 Service de Microbiologie, Unité Mobile de Microbiologie Clinique, Hôpital Européen Georges Pompidou, Assistance Publique-Hôpitaux de Paris, Paris, France; 7 Université Paris Descartes, Sorbonne Paris Cité, Paris, France; 8 INSERM, UMRS1158 Neurophysiologie respiratoire expérimentale et clinique, Sorbonne Universités, UPMC Univ Paris 06, Paris, France

**Keywords:** Sepsis, Intensive care unit, Totally implantable venous access ports

## Abstract

**Background:**

While no data support this practice, international guidelines recommend the removal of totally implanted venous access ports (TIVAPs) in patients with suspicion of TIVAP-related bloodstream infection admitted in the intensive care unit (ICU) for a life-threatening sepsis.

**Methods:**

During this multicenter, retrospective and observational study, we included all patients admitted in five ICU for a life-threatening sepsis in whom a TIVAP was removed between January 2012 and December 2014. We aimed (1) at determining the proportion of confirmed TIVAP-related infections and (2) at assessing short- and long-term survival of patients with and without TIVAP-related infections.

**Results:**

One hundred and fifty-one patients (58 ± 14 years, 62% males) were included between 2012 and 2014. TIVAP-related infections were confirmed in 68 patients (45%). Demographic characteristics were similar between patients with and without TIVAP-related infections. SOFA score on admission per point increase [odd ratio (OR), 0.86 interval confidence (IC) 95% (0.8–0.9), *p* < 0.01] and local signs of infection [OR 4.0, IC 95% (1.1–15.6), *p* = 0.04] were significantly associated with TIVAP-related infection. Patients with TIVAP-related infection had lower ICU and 6-month mortality as compared to their counterparts (9 vs. 40%, respectively, *p* < 0.01; and 50 vs. 66%, respectively, *p* = 0.04). TIVAP-related infection was significantly associated with ICU survival [OR 0.2, IC 95% (0.05–0.5), *p* < 0.01].

**Conclusions:**

TIVAP-related infection was confirmed in nearly one out of two cases of life-threatening sepsis in patients in whom it has been removed. TIVAP-related infection was associated with a good prognosis, as compared to patients with other causes of infection.

**Electronic supplementary material:**

The online version of this article (10.1186/s13613-018-0383-9) contains supplementary material, which is available to authorized users.

## Background

Totally implanted venous access ports (TIVAPs) are commonly used for patients requiring long-term or iterative treatments such as antineoplastic chemotherapy, parenteral nutrition and transfusion [1–3]. Even if TIVAPs are associated with a low risk of infection, they still remain a source of infections potentially leading to life-threatening sepsis and subsequent admission in the intensive care unit (ICU) [4, 5].

In case of tunnel or port-pocket infection, TIVAP-related bloodstream infection is obviously strongly suspected and the device should be promptly removed [6]. However, local signs of infection are frequently lacking [7–10]. International guidelines support the removal of TIVAP in case of TIVAP-related bloodstream infection, with complication like severe sepsis or/and septic shock (use of vasopressors) [6, 11], although no data support this practice. Removal of TIVAP may have deleterious consequences in critically ill patients. First, patients with TIVAP are frequently frail and exposed to uncontrolled bleeding (low platelets and coagulation disorders). Second, removal of TIVAP is a surgical procedure that may interfere with the management of the ongoing sepsis [12–15]. Eventually, removing TIVAP may defer administration of chemotherapy or specific treatments once patients are discharged from the ICU. Therefore, removal of TIVAP is an important decision that should be supported by clinical evidences, but predictive factors of TIVAP-related infections are lacking in ICU patients. Likewise, no study regarding the prognosis of TIVAP-related infections has been conducted outside the ICU [16–20]. In light with this, the present study was designed to address three main objectives: (1) to determine the proportion of confirmed TIVAP-related infections in a population of patients admitted in the ICU in whom a TIVAP was removed for life-threatening sepsis, (2) to identify predictive factors of confirmed TIVAP infection in patients admitted to the ICU and (3) to assess short- and long-term outcome of patients with TIVAP-related infection and compare them with their counterparts in whom TIVAP was removed without confirmation of infection.

## Patients and methods

This retrospective, multicenter, observational study was conducted in five ICU located in academic hospitals. The study period extended from January 2012 through December 2014. The Institutional Review Board of the French Intensive Care Society approved the study (CE SRLF15-52).

### Selection of patients

Using clinical microbiology laboratory databases, we identified retrospectively all the patients admitted in participating ICU in whom a TIVAP was removed during the ICU stay and sent to the microbiology laboratory. Each patient’s record was analyzed by two investigators (ML and MD) and those patients who fulfilled the following criteria were entered into the study: (1) age > 18 years, (2) sepsis, severe sepsis or septic shock, defined according to criteria of the Surviving Sepsis Campaign’s definition [21], as the main reason for TIVAP removal. Of notice, peripherally inserted central catheters and surgically inserted long-term central venous catheters others than TIVAP were not considered for the study. In addition, patients with TIVAP removed before ICU admission or for another reason than sepsis (thrombosis, uselessness) were also not included in the study.

### Data collection

The following data were extracted from each patient’s medical record: age, gender, clinical and biological variables on admission. Simplified Acute Physiology Score (SAPS) 2 [22] and Sequential Organ Failure Assessment (SOFA) [23] were calculated upon ICU admission. Predisposing risk factors for TIVAP-related infections were also collected: immunosuppression status (i.e., hematological malignancies, solid organ transplant, recent antineoplastic chemotherapy for cancer or HIV infection), time since TIVAP insertion, main indication of the TIVAP (antineoplastic chemotherapy, parenteral nutrition), date of last antineoplastic chemotherapy and delay between ICU admission and removal of the device. We also looked through each patient’s record for local signs of infection (induration or erythema, warmth and pain or tenderness along the tract of a catheterized vein) whenever it was described and general sign of infection (fever defined as temperature ≥ 38°3, hypothermia defined as temperature < 36°, hypotension defined as systolic blood pressure < 90 mmHg or mean blood pressure < 65 mmHg). Advanced life support measures taken during the ICU stay (mechanical ventilation and vasopressors) and antibiotics regimens were recorded for each patient. Microbiological data were collected as follows: positive culture of TIVAP catheter tip or port reservoir, positive blood culture from the TIVAP and from a peripheral vein with the differential time to positivity. We also collected information regarding use of appropriate antibiotic in initial regimen (antibiotics with in vitro activity against the infecting agent). Finally, we recorded length of ICU stay, time spent under invasive mechanical ventilation and vasopressors. Mortality was determined in the ICU, at 28 days and 6 months after ICU admission.

### Definitions

Culture of TIVAP was considered as positive if the tip or port reservoir (indoor or outdoor) was positive. Positivity of TIVAP tip culture was defined according to the same modality across all the microbiological laboratories of participating centers. It was defined on blood agar plate by quantitative method after vortexing or sonication (taking into account pathogens present in their inner or outer surfaces) with a cutoff of ≥ 1000 colony-forming units (CFU)/mL [24–26]. Growth of < 1000 CFU/mL from a catheter by quantitative method was considered as catheter contamination. TIVAP box culture was performed according to each clinical microbiology laboratory-own protocol, such as immersion of the case in broth and then sowing on blood agar plate or chocolate plate, needle puncture and aspiration of the case contents then sowing, or swab from outside the case then sowing. In all these techniques, a qualitative culture was performed.

Definition of TIVAP-related infection was adapted from the Infectious Diseases Society of America (IDSA) guidelines [6] as one of the following conditions:

1. TIVAP-related bloodstream infection, defined as (1) a positive culture of the TIVAP (catheter tip or reservoir’s port) associated with a positive peripheral blood culture with the same microorganism (same species and same antibiotic susceptibility testing) or (2) a differential time to positivity of a blood culture drawn from the catheter versus from a peripheral vein (positivity of the catheter blood sample at least 2 h before the peripheral blood sample) [27, 28];
2. Local or general (fever ≥ 38°3 or < 36° and chills) signs of infection, positive culture of TIVAP (catheter tip or the reservoir’s port) and regression of clinical signs of infection after TIVAP removal despite a negative peripheral blood culture.

We also included patients who did not meet the two above conditions but who had positive blood culture without other suspected infection and regression of clinical signs of infection after TIVAP removal despite negative culture of TIVAP (catheter tip or the reservoir’s port).

Exclusive TIVAP-related infection was defined by TIVAP-related infection that was not associated with any other documented source of infection among lower respiratory, digestive or urinary tract infection.

### Statistical analysis

Continuous variables are expressed as mean ± standard deviation or median (interquartile range). Categorical variables are expressed as number and relative frequencies. Patients were categorized a posteriori into two groups according to microbiologic findings: patients with or without TIVAP-related infection. Continuous variables were tested for normality using the Shapiro–Wilk test. Gaussian variables were compared using a *t* test and non-normally distributed variables using a Mann–Whitney test. Categorical variables were compared with Chi-square test. The primary endpoint was the prevalence of TIVAP-related infection. A stepwise logistic regression analysis was performed to identify variables associated with TIVAP-related infection and with ICU and 28-day mortality. Variables found to have univariate association (*p* < 0.05) with the outcome of interest were considered in the final model. Kaplan–Meier survival curves for patients with and without TIVAP-related infections were computed for 6-month mortality. For all final comparisons, a two-tailed *p* value less than or equal to 0.05 was considered statistically significant. The statistical analysis was performed with SAS statistical V9.3 software (SAS Institute Inc., Cary, NC, USA).

## Results

### Characteristics upon ICU admission

Over the study period, 151 patients met the inclusion criteria and were retained in the analysis (see flowchart, Additional file 1: Figure S1). Main characteristics of the patients are presented in Table 1. One hundred and forty-eight (98%) patients were immunosuppressed, and 50 patients (33%) had neutropenia. Antineoplastic chemotherapy was administrated in 131 patients (87%) in the last 6 months, and 18 (12%) of the patients were receiving parenteral nutrition. Severe sepsis was present in 48 patients (32%) and septic shock in 93 (62%).

**Table 1:** Clinical characteristics and laboratory features upon intensive care unit admission

Characteristic	All *n* = 151	TIVAP-related infection *n* = 68	No TIVAP-related infection *n* = 83	*p* value
Age, year	58 ± 14	57 ± 14	58 ± 14	0.58
Female gender, *n* (%)	58 (38)	30 (44)	28 (34)	0.19
Transfer from the emergency room, *n* (%)	47 (31)	21 (31)	26 (31)	1.00
Transfer from the ward, *n* (%)	104 (69)	47 (69)	57 (69)	1.00
SAPS2	52 ± 17	47 ± 15	56 ± 17	< 0.01
SOFA	9 ± 4	7 ± 4	10 ± 4	< 0.01
TIVAP-related infection risk factors, *n* (%)				
Immunosuppression	148 (98)	66 (97)	82 (99)	0.44
Hematological malignancies	72 (48)	28 (41)	44 (53)	0.14
Solid organ cancer	71 (47)	35 (51)	36 (43)	0.32
Metastatic cancer	44 (29)	24 (35)	20 (24)	0.13
Recent chemotherapy (< 6 months)	131 (87)	58 (85)	73 (88)	0.63
Parenteral nutrition	18 (12)	14 (21)	4 (5)	< 0.01
Clinical signs				
Temperature < 36 or ≥ 38.3 °C, *n* (%)	111 (74)	55 (81)	56 (67)	0.08
Systolic blood pressure, mmHg	98 ± 27	97 ± 29	99 ± 25	0.54
Mean blood pressure, mmHg	69 ± 20	69 ± 23	70 ± 18	0.77
Glasgow Score Scale	13 ± 3	14 ± 3	13 ± 4	0.04
Local sign of infection, *n* (%)	15 (10)	12 (18)	3 (4)	< 0.01
Biological signs				
White blood cells < 1 Giga/l, *n* (%)	50 (33)	19 (28)	31 (37)	0.22
Platelet counts, Giga/l	116 ± 113	124 ± 99	110 ± 123	0.45
Prothrombin time, %	64 ± 17	69 ± 17	60 ± 17	< 0.01
Serum creatinine, μmol/l	142 ± 119	126 ± 113	155 ± 123	0.13
Bicarbonate, mmol/l	20 ± 5	21 ± 5	20 ± 6	0.05
Arterial lactate, mmol/l	3.4 ± 3.2	3.3 ± 3.0	3.6 ± 3.3	0.61

Categorical variables are expressed as no. (%) and continuous variables as mean ± SD

*SAPS2* Simplified Acute Physiology Score, *SOFA* Sepsis-Related Organ Failure Assessment, *TIVAP* totally implanted venous access port

### Proportion and features of TIVAP-related infection

TIVAP-related infection was found in 68 patients (45%). Among these 68 patients, the diagnosis of TIVAP-related infection was retained because of (1) TIVAP-related bloodstream infection with the association of a positive peripheral blood culture with a positive culture of the TIVAP (tip or reservoir) in 33 patients (48%) or a differential time to positivity of a blood culture drawn from the catheter versus from a peripheral vein in 11 patients (16%); (2) a positive culture of the TIVAP and the regression of clinical signs of infection after TIVAP removal in 12 (18%) patients (negative peripheral blood culture) or (3) a positive blood culture associated with favorable outcome after TIVAP removal in 12 (18%) patients (negative tip or reservoir culture of the TIVAP). A growth of 100 CFU/mL of *Staphylococcus epidermidis* was found in two patients who were classified as catheter contamination (without TIVAP-related infection).

Regarding microbiological findings, TIVAP-related infections were associated with 53% (36/68) of Gram-negative rods, 44% (30/68) of Gram-positive cocci and 7% (5/68) of *Candida* sp. (see Table 2). Sixty-nine percent of patients with TIVAP-related infection had no other focus of infection and were subsequently classified as exclusive TIVAP-related infection (see Additional file 1: Table S1). Among patients with infectious other than TIVAP-related infections, 65% had positive microbiological samples (see Table 3; Additional file 1: Table S2).

**Table 2:** Microbiological findings in patients with totally implanted venous access ports-related infections and association with 28-day mortality (univariate analysis)

	All *n* = 68	Alive *n* = 54	Dead *n* = 14	*p* value
Gram-negative bacilli, *n* (%)	36 (53)	32 (59)	4 (29)	0.07
*Enterobacteriaceae*	27 (40)	25 (46)	2 (14)	0.03
*Escherichia coli*	8 (12)	8 (15)	0 (0)	0.19
*Klebsiella pneumoniae*	7 (10)	6 (11)	1 (7)	0.99
*Enterobacter cloacae*	7 (10)	7 (13)	0 (0)	0.33
Other *Enterobacteriaceae*	6 (9)	5 (9)	1 (7)	0.99
*Pseudomonas aeruginosa*	8 (12)	6 (11)	2 (14)	0.99
*Stenotrophomonas maltophilia*	1 (2)	1 (2)	0 (0)	0.99
*Acinetobacter* sp.	1 (2)	1 (2)	0 (0)	0.99
Gram-positive cocci, *n* (%)	30 (44)	21 (39)	9 (64)	0.13
*Staphylococcus aureus*	9 (13)	5 (9)	4 (29)	0.09
Coagulase-negative staphylococci	19 (28)	15 (28)	4 (29)	0.99
*Enterococcus* sp.	2 (3)	1 (2)	1 (7)	0.37
Other Gram-positive cocci	1(2)	1 (2)	0 (0)	0.99
*Candida* sp., *n* (%)	5 (7)	2 (4)	3 (21)	0.06
Polymicrobial, *n* (%)	6 (9)	4 (7)	2 (14)	0.59

Data are expressed as *n* (%)

**Table 3:** Microbiological findings in patients without TIVAP (totally implanted venous access port)-related infections and association with 28-day mortality (univariate analysis)

	All *n* = 83	Alive *n* = 43	Dead *n* = 40	*p* value
Gram-negative bacilli, *n* (%)	34 (41)	17 (40)	17 (43)	0.83
*Enterobacteriaceae*	26 (31)	15 (35)	11 (28)	0.49
*Escherichia coli*	14 (17)	7 (16)	7 (18)	0.99
*Klebsiella pneumoniae*	7 (9)	4 (9)	3 (8)	0.99
*Enterobacter cloacae*	3 (4)	2 (5)	1 (3)	0.99
Other *Enterobacteriaceae*	9 (11)	7 (16)	2 (5)	0.16
*Pseudomonas aeruginosa*	6 (7)	1 (2)	5 (13)	0.10
*Acinetobacter* sp.	2 (3)	1 (2)	1 (3)	0.99
Gram-positive cocci, *n* (%)	22 (27)	8 (19)	14 (35)	0.13
*Staphylococcus aureus*	4 (5)	2 (5)	2 (5)	0.99
Coagulase-negative staphylococci	4 (5)	0 (0)	4 (10)	0.05
*Enterococcus* sp.	10 (12)	3 (7)	7 (18)	0.18
Other Gram-positive cocci	5 (6)	3 (7)	2 (5)	0.99
Other bacteria, *n* (%)	4 (5)	4 (9)	0 (0)	0.12
*Candida* sp., *n* (%)	8 (10)	4 (9)	4 (10)	0.99
Polymicrobial, *n* (%)	16 (19)	7 (16)	9 (23)	0.58

Data are expressed as *n* (%)

### Characteristics of the patients according to the presence or absence of TIVAP-related infections

Demographic characteristics of patients with and without TIVAP-related infections were not different (Table 1). Parenteral nutrition was more frequent in patients with TIVAP-related infections. In addition, SAPS2 and SOFA score were higher in patients with infectious other than TIVAP-related infections as compared to their counterparts. Local signs of infection were present in 18% of the patients with a TIVAP-related infection versus 4% for their counterparts (*p* < 0.01). There was no difference regarding the presence of neutropenia upon admission between patients with TIVAP-related infection and patients without.

By multivariate logistic regression analysis, two factors were independently associated with TIVAP-related infection: SOFA score upon admission per point increase [odd ratio (OR) 0.86 interval confidence (IC) 95% (0.80–0.90), *p* < 0.01] and local signs of infection [OR 4.0 IC 95% (1.1–15.6), *p* = 0.04].

### Therapeutic management and outcome

**Fig. 1:**
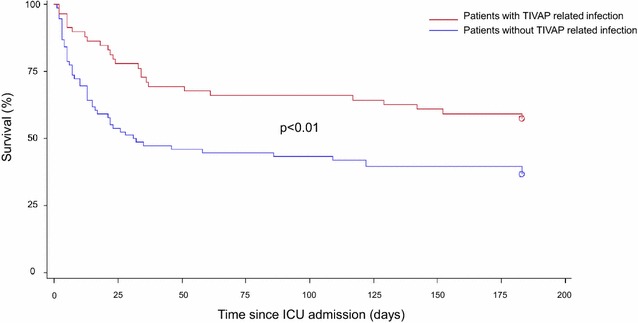
Kaplan–Meier survival curve with 6-month mortality in patients with and without totally implanted venous access port (TIVAP)-related infections

Patients with infections other than TIVAP-related infections received more vasopressors and were more likely to require mechanical ventilation than their counterparts (Table 4). There was no significant difference in terms of antibiotics management between both groups. The duration of ICU stay was similar between patients with and without TIVAP-related infections. Overall ICU mortality was 26%. Patients with TIVAP-related infection had lower ICU, 28-day and 6-month mortality as compared to their counterparts (Table 4; Fig. 1). In addition, patients with exclusive TIVAP-related infection had a lower ICU mortality as compared to patients who had TIVAP-related infection and another focus of infection: 2% (1/47) versus 24% (5/21), respectively (*p* < 0.01).

**Table 4:** Therapeutic management and outcome of patients with and without TIVAP (totally implanted venous access port)-related infection

Characteristic	All patients *n* = 151	TIVAP-related infections *n* = 68	No TIVAP-related infections *n* = 83	*p* value
Time between ICU admission and TIVAP withdrawal, days	1 (0–2)	1 (0–1)	1 (0–2)	0.09
Antibiotics				
Beta lactam antibiotic, *n* (%)	143 (95)	63 (93)	80 (96)	0.49
Glycopeptide/linezolid, *n* (%)	78 (52)	38 (57)	40 (48)	0.29
Use of MV, *n* (%)	74 (49)	24 (35)	50 (60)	< 0.01
MV duration, days	0 (0–5)	0 (0–3)	2 (0–7)	0.26
Vasopressors, *n* (%)	103 (68)	37 (55)	66 (80)	< 0.01
Vasopressors, days	2 (0–3)	1 (0–2)	2 (1–4)	0.04
ICU length of stay, days	5 (3–10)	4 (3–6)	6 (3–12)	0.40
ICU mortality, *n* (%)	39 (26)	6 (9)	33 (40)	< 0.01
28-day mortality, *n* (%)	54 (36)	14 (20.5)	40 (48)	< 0.01
6-Month mortality, *n* (%)	89 (59)	34 (50)	55 (66)	0.04

Categorical variables are expressed as no. (%) and continuous variables as median (interquartile range)

*TIVAP* totally implanted venous access port, *ICU* intensive care unit, *MV* mechanical ventilation

Table 5 displays the variables associated with 28-day mortality, and Tables 2 and 3 show microbiological findings associated with 28-day mortality among patients with and without TIVAP-related infections, respectively. By multivariate logistic regression analysis, three factors were independently associated with higher 28-day mortality: SOFA at admission per point increase [OR 1.3 IC 95% (1.1–1.6), *p* < 0.01], the use of mechanical ventilation [OR 11 IC 95% (2.8–41.2), *p* < 0.01] and hematological malignancies [OR 3.2 IC 95% (1.1–9.1), *p* = 0.03]. One factor, TIVAP-related infection, was independently associated with lower 28-day mortality [OR 0.2 IC 95% (0.1–0.7), *p* = 0.02].

**Table 5:** Variables associated with 28-day mortality

	All *n* = 151	Alive *n* = 97	Dead *n* = 54	*p* value
Age, year	58 ± 14	57 ± 15	59 ± 13	0.40
Female gender, *n* (%)	58 (38)	41 (43)	17 (32)	0.71
SAPS2	52 ± 17	47 ± 13	62 ± 19	< 0.01
SOFA	9 ± 4	7 ± 3.5	11 ± 4.5	< 0.01
TIVAP-related infection	68 (45)	55 (57)	13 (24)	< 0.05
TIVAP-related infection risk factors, *n* (%)				
Immunosuppression	148 (98)	97 (100)	51 (95)	0.99
Hematological malignancies	72 (48)	43 (44)	29 (54)	< 0.05
Solid organ cancer	71 (47)	52 (54)	19 (35)	0.12
Metastatic cancer	44 (29)	31 (32)	13 (24)	0.55
Recent chemotherapy (< 6 months)	131 (87)	88 (91)	43 (80)	0.85
Parenteral nutrition	18 (12)	13 (14)	5 (9)	0.34
Initial presentation				
Systolic blood pressure, mmHg	98 ± 27	98 ± 27	101 ± 28	0.55
Mean blood pressure, mmHg	69 ± 20	69 ± 19	72 ± 24	0.44
Glasgow Score Scale	13 ± 3	14 ± 3	13 ± 3	0.20
White blood cells, Giga/l	7.6 ± 13.2	7.6 ± 15.6	7.6 ± 10.2	0.98
Platelet counts, Giga/l	116 ± 113	128 ± 110	91 ± 114	< 0.05
Prothrombin time, %	64 ± 17	68 ± 15	55 ± 19	< 0.01
Serum creatinine, μmol/l	142 ± 119	125 ± 106	176 ± 137	0.01
Bicarbonate, mmol/l	20 ± 5	21 ± 6	19 ± 5	< 0.01
Arterial blood lactate, mmol/l	3.4 ± 3.2	2.7 ± 2.2	5 ± 4	< 0.01
Treatments				
Time between ICU admission and device withdrawal, days	1.8 ± 3.5	1.6 ± 2.8	2.2 ± 4.5	0.35
Use of MV, *n* (%)	74 (49)	37 (38)	37 (69)	< 0.01
MV duration, days	4 ± 7	4 ± 7	5 ± 5	0.32
Use of vasopressors, *n* (%)	103 (68)	62 (64)	41 (76)	< 0.01
Vasopressors duration, days	3 ± 4	2 ± 3	4 ± 5	< 0.01
ICU stay, days	8 ± 9	9 ± 10	8 ± 7	0.53

Categorical variables are expressed as no. (%) and continuous variables as mean ± SD

*ICU* intensive care unit, *SOFA* Sepsis-Related Organ Failure Assessment, *SAPS2* Simplified Acute Physiology Score, *TIVAP* totally implanted venous access port, *MV* mechanical ventilation

Additional file 1: Table S3 displays the variables associated with ICU mortality. By multivariate logistic regression analysis, two factors were independently associated with higher ICU mortality: SOFA at admission per point increase [OR 1.4 IC 95% (1.2–1.7), *p* < 0.01] and the use of mechanical ventilation [OR 14.0 IC 95% (3.6–56.0), *p* < 0.01]. One factor, TIVAP-related infection, was independently associated with lower ICU mortality [OR 0.16 IC 95% (0.05–0.5), *p* < 0.01].

## Discussion

This study is seemingly the first reporting characteristics and outcome of patients admitted for a life-threatening sepsis in the ICU whose TIVAP has been removed. Our findings can be summarized as follows: (1) TIVAP-related infection was confirmed in almost half of the patients; (2) except for a low value of SOFA score and local signs of infection, no other variable was found to be independently associated with TIVAP-related infection upon admission; and (3) patients with TIVAP-related infection had a better prognosis than patients with other source of infection.

### Proportion of TIVAP-related infection

The proportion of patients in whom a TIVAP-related infection was confirmed was 45%. Being the first study addressing this issue in the ICU, our results are hardly comparable. Indeed, most of previous studies were conducted outside the ICU [4, 5, 10, 17, 19, 20, 29]. For instance, in two studies [8, 9], among patient whose TIVAP was removed because of a suspected infection, TIVAP-related infection was confirmed in two thirds of the patients (19/29 and 15/23, respectively). Based on our findings, the remaining key question is whether this 45% rate is high enough to justify TIVAP removal as supported by international guidelines [6] or whether these recommendations should be questioned. A higher rate (> 80%) would have made clearly acceptable the TIVAP removal (and all its consequences), whereas a lower rate (< 20%) would have discouraged current practices in term of potential harmful effects. While this 45% rate of TIVAP-related infection could be considered disappointing, it might represent the lower bounder of the proportion of patients with TIVAP-related infection. Indeed, all our patients received antibiotics prior ICU admission, a condition that could easily lead to false-negative microbiological findings [30]. As a matter of fact, this could have artificially underestimated the rate of patients with confirmed TIVAP-related infections. A randomized controlled trial might be necessary to definitively support the removal of TIVAP in case of severe sepsis or septic shock.

Another important finding is that a concomitant infection focus was evidenced in approximately one-third of patients with TIVAP-related infection. In half of these cases, the same pathogen was responsible (Additional file 1: Table S1). Whether this focus was the primary source or an hematogenous seeding from the TIVAP-related infection is unknown. This finding points out the importance of an extensive investigation even though a TIVAP infection is identified.

### Factors associated with TIVAP-related infection

While a 45% rate of confirmed TIVAP-related infection may be acceptable, identifying associated factors of TIVAP-related infection upon admission would be helpful to prevent patients from undue procedures. Unfortunately, our study failed at providing new insightful clues in this purpose. Immunosuppression, a known risk factor for infection [4, 31–33], was equally present in both groups. By contrast, the use of parenteral nutrition, another recognized risk factor for TIVAP-related infection [34–36], was used more frequently in patients with TIVAP-related infection, but this finding remained infrequent and was not confirmed in multivariate analysis. While local signs of infection have been reported to be present from 7 to 33% of the cases [8–10, 17, 20], in our patient series, only few had local sign of infection (18%). But it’s important to specify that information about local signs of infection is difficult to collect in a retrospective study. They are not systematically looked for and their identification may vary between healthcare providers when clear definitions are not used. Interestingly, it was independently associated with TIVAP-related infection in our study.

### TIVAP-related infections: impact on prognosis

While ICU mortality of sepsis is still high, up to 20% in latest publications [37–39], it reaches even higher rates in immunocompromised patients, between 40 and 60% [40]. In the present study, we report a lower mortality: 26% for ICU mortality and 36% at 28 days. While expected mortality (predicted by SAPS2 score) was around 40%, patients with TIVAP-related infections had a much lower mortality (9%). Moreover, ICU mortality was 2% in patients with exclusive TIVAP-related infection (no other infection focus). TIVAP-related infection was significantly associated with highest ICU and 28-day survival, whereas high SOFA score upon admission and the need for mechanical ventilation were significantly associated with ICU mortality and at 28 days. The low mortality observed in patients with exclusive TIVAP-related infections may be explained by the strong experience in the management of immunocompromised patients of participating ICUs. As a matter of fact, the precociousness of source control was probably a major driver of such a favorable outcome. In agreement with other studies, neutropenia was not significantly related to mortality [41–44]. Interestingly, patients who had both TIVAP-related infections and another focus had a worst prognosis than patients who had only TIVAP-related infection. In light with this, our study provides an important message for physicians managing immunosuppressed patients with severe presentation (septic shock, need for mechanical ventilation). While admission in the ICU of such patients is sometimes questioned, our findings suggest that in case of TIVAP-related infection, those patients have a short ICU stay and good prognosis. Likewise, our findings suggest that in patients admitted for a suspected TIVAP-related infection who do not recover shortly, this diagnosis should be questioned.

### Limits

While reporting the first cohort of TIVAP-related infections in the ICU, this retrospective study has some limitations. As we obtained data from patient’s medical charts, we cannot pretend to be exhaustive in data collection. Important information such as presence of thrombosis or endocarditis associated with TIVAP-related infection would have been interesting, but not all patients had a Doppler ultrasonography at time of admission. Then, it is likely that all patients with TIVAP admitted in participating ICU were not enrolled in the study since the main inclusion criteria was TIVAP removal. As a matter of fact, our inclusion criteria could have biased the true incidence of TIVAP-related infection toward overestimation. Due to the retrospective nature of the study, it was not possible to systematically identify patients who may have been admitted in our ICUs with a TIVAP in place that was not removed. The fact that positive blood cultures were available prior TIVAP removal may have also influenced clinicians’ decision. However, ICUs participating in the study had homogeneous practices regarding admission policy of immunocompromised patients and the decision to remove TIVAP in case of life-threatening sepsis. Last, the definition of TIVAP-related infection was extended to patients with positive blood culture without other suspected infection (*n* = 12) and regression of clinical signs of infection after TIVAP removal despite negative culture of TIVAP (catheter tip or the reservoir’s port). Since all our patients received antibiotics prior TIVAP removal, a condition that could fairly lead to misclassification, we estimated legitimate to consider these twelve patients as having TIVAP-related infection.

## Conclusion

In almost one out of two cases, TIVAP-related infection was evidenced in immunosuppressed patients admitted in the ICU for sepsis and in whom the device has been removed. With the exception of local signs of infection, no other associated factor could have been identified. TIVAP-related infection was associated with a good prognosis, as compared to patients with other causes of infection.

## Additional file

**Additional file 1: Table 1.** Complementary microbiological findings in patients with TIVAP (totally implanted venous-access ports) related infections. **Table E2.** Complementary microbiological findings in patients without TIVAP (totally implanted venous-access ports) related infections. **Table E3.** Variables associated with ICU mortality (univariate analysis). **Figure E1.** Flow chart of the patients. ICU: intensive care unit; TIVAP: totally implanted venous access ports.

## Abbreviations

TIVAP: totally implanted venous access port
ICU: intensive care unit
SAPS II: Simplified Acute Physiology Score II
SOFA: Sequential Organ Failure Assessment
HIV: human immunodeficiency virus
IDSA: Infectious Diseases Society of America
CFU: colony-forming unit
OR: odd ratio
CI: confidence interval
