# Supplementary material for: Removal of totally implanted venous access ports for suspected infection in the intensive care unit: a multicenter observational study
Source: Ann Intensive Care. 2018 Mar 27;8:41. doi: 10.1186/s13613-018-0383-9 (PMC5874227; doi:10.1186/s13613-018-0383-9)
Supplement: Supplementary file 1 — Additional file 1: Table 1. Complementary microbiological findings in patients with TIVAP (totally implanted venous-access ports) related infections. Table E2. Complementary microbiological findings in patients without TIVAP (totally implanted venous-access ports) related infections. Table E3. Variables associated with ICU mortality (univariate analysis). Figure E1. Flow chart of the patients. ICU: intensive care unit; TIVAP: totally implanted venous access ports. [file 13613_2018_383_MOESM1_ESM.docx]

Removal of Totally Implanted Venous Access Ports for Suspected Infection in the Intensive Care Unit: a multicenter observational study

Marie Lecronier, Sandrine Valade, Naike Bigé, Nicolas de Prost, Damien Roux, David Lebeaux, Eric Maury, Elie Azoulay, Alexandre Demoule, Martin Dres on behalf of the GrrrOH (Group for Research in Respiratory Intensive Care Onco-Hematology).

**Supplementary**

**Table S1.** Complementary microbiological findings in patients with TIVAP (totally implanted venous-access ports) related infections.

**Table S2**. Complementary microbiological findings in patients without TIVAP (totally implanted venous-access ports) related infections.

**Table S3.** Variables associated with ICU mortality (univariate analysis).

**Figure S1.** Flow chart of the patients. ICU: intensive care unit; TIVAP: totally implanted venous access ports.

**Table S1**: Complementary microbiological findings in patients with TIVAP (totally implanted venous-access ports) related infections.

|  | **TIVAP-related infections**  **n = 68** |
| --- | --- |
| **Microbiological documentation** |  |
| Peripheral bloodstream positive culture, n (%) | 54 (79) |
| TIVAP positive culture (tip or the reservoir’s port), n (%) | 56 (82) |
| Time between first bloodstream positive culture and TIVAP removal, days | 1.1 ± 1.2 |
| Positive bloodstream culture before TIVAP removal, n (%) | 30 (44) |
| Use of appropriate antibiotic in initial regimen, n (%) | 50 (74) |
| **Type of infection** |  |
| Exclusive TIVAP-related infection, n (%) | 47 (69) |
| TIVAP-related infection associated with another infection, n (%) | 21 (31) |
| Infection focus |  |
| Digestive tract | 8/21 (38) |
| Low respiratory tract | 7/21 (33) |
| Urinary tract | 1/21 (5) |
| Endocarditis | 1/21 (5) |
| Isolated Bloodstream | 4/21 (19) |
| Microorganisms, n (%) |  |
| Same bacteria as TIVAP-related infection | 10/21 (48) |
| Different bacteria from TIVAP-related infection | 11/21 (52) |

Categorical variables are expressed as No. (%) and continuous variables as mean ± SD.

TIVAP: totally implanted venous access ports.

**Table S2**. Complementary microbiological findings in patients without TIVAP (totally implanted venous-access ports) related infections.

|  | **Patients without TIVAP related infection**  **n = 83** |
| --- | --- |
| **No microbiologic data** | 29 (35) |
| **Type of infection, n (%)** |  |
| Low respiratory tract infection | 40 (48) |
| Digestive tract infection | 22 (27) |
| Urinary tract infection | 7 (9) |
| Others | 15 (18) |

TIVAP: totally implanted venous access ports.

**Table S3.** Variables associated with ICU mortality (univariate analysis).

| **Characteristic** | | **All**  **n = 151** | | **Alive**  **n = 112** | | **Dead**  **n = 39** | | **p value** | |
| --- | --- | --- | --- | --- | --- | --- | --- | --- | --- |
| Age, year | | 58 ± 14 | | 57 ± 14 | | 59 ± 13 | | 0.53 | |
| Female gender, n (%) | | 58 (38) | | 44 (39) | | 14 (36) | | 0.71 | |
| SAPS2 | | 52 ± 17 | | 47 ± 14 | | 66 ± 18 | | <0.01 | |
| SOFA | | 9 ± 4 | | 7 ± 4 | | 12 ± 4 | | <0.01 | |
| **TIVAP-related infection** 68 (45) 62 (55) | | | | | | 6 (15) | | <0.01 | |
| **TIVAP-related infection risk factors,** **n (%)** | | | | | |  | |  | |
| Immunosuppression | | 148 (98) | | 110 (98) | | 38 (98) | | 0.76 | |
| Hematological malignancies | | 72 (48) | | 46 (41) | | 26 (67) | | <0.01 | |
| Solid organ cancer | | 71 (47) | | 60 (54) | | 11 (28) | | <0.01 | |
| Metastatic cancer | | 44 (29) | | 38 (34) | | 6 (16) | | 0.03 | |
| Recent chemotherapy (< 6 months) | | 131 (87) | | 98 (88) | | 33 (85) | | 0.65 | |
| Parenteral nutrition | | 18 (12) | | 15 (14) | | 3 (8) | | 0.34 | |
| **Initial presentation** | |  | |  | |  | |  | |
| Systolic blood pressure, mmHg | | 98 ± 27 | | 99 ± 29 | | 97 ± 22 | | 0.77 | |
| Mean blood pressure, mmHg | | 69 ± 20 | | 70 ± 22 | | 68 ± 16 | | 0.55 | |
| Glasgow score scale | | 13 ± 3 | | 14 ± 3 | | 12 ± 4 | | 0.04 | |
| White blood cells, Giga/l | | 7.6 ± 13.2 | | 8.0 ± 14.4 | | 6.5 ± 9.2 | | 0.27 | |
| Platelet counts, Giga/l | | 116 ± 113 | | 129 ± 113 | | 79 ± 103 | | 0.01 | |
| Prothrombin time, % | | 64 ± 17 | | 68 ± 15 | | 51 ± 17 | | <0.01 | |
| Serum creatinine, μmol/l | | 142 ± 119 | | 126 ± 103 | | 188 ± 150 | | <0.01 | |
| Bicarbonate, mmol/l | | 20 ± 5 | | 21 ± 6 | | 17 ± 5 | | <0.01 | |
| Arterial blood lactate, mmol/l | | 3.4 ± 3.2 | | 2.8 ± 2.3 | | 5.2 ± 4.5 | | <0.01 | |
| **Treatments** | |  | |  | |  | |  | |
| Time between ICU admission and device withdrawal, days | | 1.8 ± 3.5 | | 1.5 ± 2.7 | | 2.6 ± 5.1 | | 0.10 | |
| Use of MV, n (%) | | 74 (49) | | 38 (34) | | 36 (92) | | <0.01 | |
| MV duration, days | | 4 ± 7 | | 23 ± 5 | | 8 ± 9 | | <0.01 | |
| Use of vasopressors, n (%) | | 103 (68) | | 66 (59) | | 37 (95) | | <0.01 | |
| Vasopressors duration, days | | 3 ± 4 | | 2 ± 3 | | 5 ± 5 | | <0.01 | |
| ICU stay, days | | 8 ± 9 | | 8 ± 9 | | 10 ±10 | | 0.10 | |

Categorical variables are expressed as No. (%) and continuous variables as mean ± SD

ICU, intensive care unit; SOFA, Sepsis related Organ Failure Assessment; SAPS2, simplified acute physiology score; TIVAP, totally implanted venous-access ports, MV, mechanical ventilation.

**Figure S1.**

**
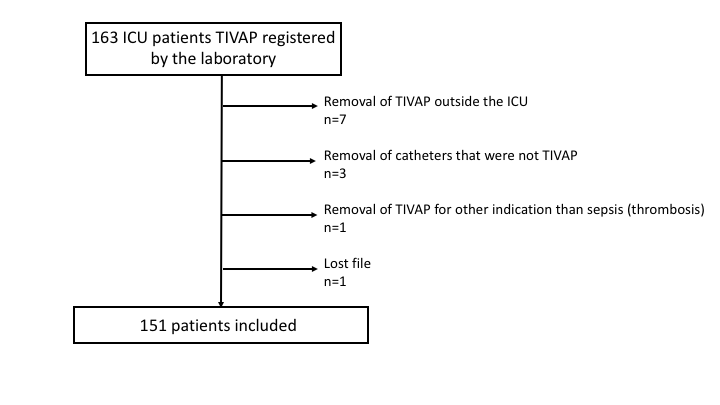
**
